# Comment on Long-Term Normothermic Machine Preservation of Partial Livers: First Experience With 21 Human Hemilivers

**DOI:** 10.1097/AS9.0000000000000124

**Published:** 2022-02-03

**Authors:** Hynek Mergental, Barney T. F. Stephenson, Richard W. Laing, Paolo Muiesan, M. Thamara P. R. Perera, Simon C. Afford, Darius F. Mirza

**Affiliations:** From the * Liver Unit, Queen Elizabeth Hospital, University Hospitals Birmingham NHS Foundation Trust (UHBFT), Birmingham, United Kingdom; † National Institute for Health Research (NIHR), Birmingham Biomedical Research Centre, University of Birmingham and UHBFT, Birmingham, United Kingdom; ‡ Centre for Liver and Gastrointestinal Research, Institute of Immunology and Immunotherapy, University of Birmingham, Birmingham, United Kingdom.

## Dear Editor:

We read the publication by Mueller et al with great interest.^1^ The Zurich group’s innovative research of partial liver perfusion sustained for up to 7 days opens many opportunities. The described model combines sophisticated hepatic surgery with new insights regarding the physiology of livers perfused *ex situ,* and authors foresee its use in advanced cancer treatment including by partial liver autotransplantation.

Our team has used normothermic machine perfusion strategies as a platform for mechanistic research. In our setting, although we were unable to modify the liver resection technique to live donor-like dissection, our development objective was to establish models that are more applicable to wider research community. We present our experience focused on approaches to restore vascular inflow to partial human hemi livers, with vascular reconstruction for right and left hepatectomy specimens, as well as full-size diseased livers obtained from transplant recipient hepatectomies. Key perfusion characteristics hopefully act as a reference point to guide others in development of such research perfusion protocols.

For the partial liver perfusion model, the important planning steps were the assessment of preoperative CT scans and discussion with the surgeon regarding the minor alterations of the hepatectomy procedure with respect to hepatic pedicle transection. Following failures to reconstruct hepatic arteries divided within parenchyma using stapler devices, we targeted operations where we envisaged: The right or left hepatic artery could be safely separated and divided outside the parenchyma; parenchyma transection likely to be performed without a Pringle’s maneuver; and favorable tumor location to obtain representative tissue samples before commencing normothermic perfusion. n recipient hepatectomies, the explanted specimen was more standardized, and we targeted procedures without history of transarterial chemoembolization, with patent portal vein, and transplantations without long warm ischemia caused by routine creation of temporary portocaval shunt. For both types, the artery stump was flushed with heparinized saline before being closed with a bulldog clamp. If feasible, the liver was flushed with cold preservation solution immediately after being handed over by the operating team.

**FIGURE 1.**
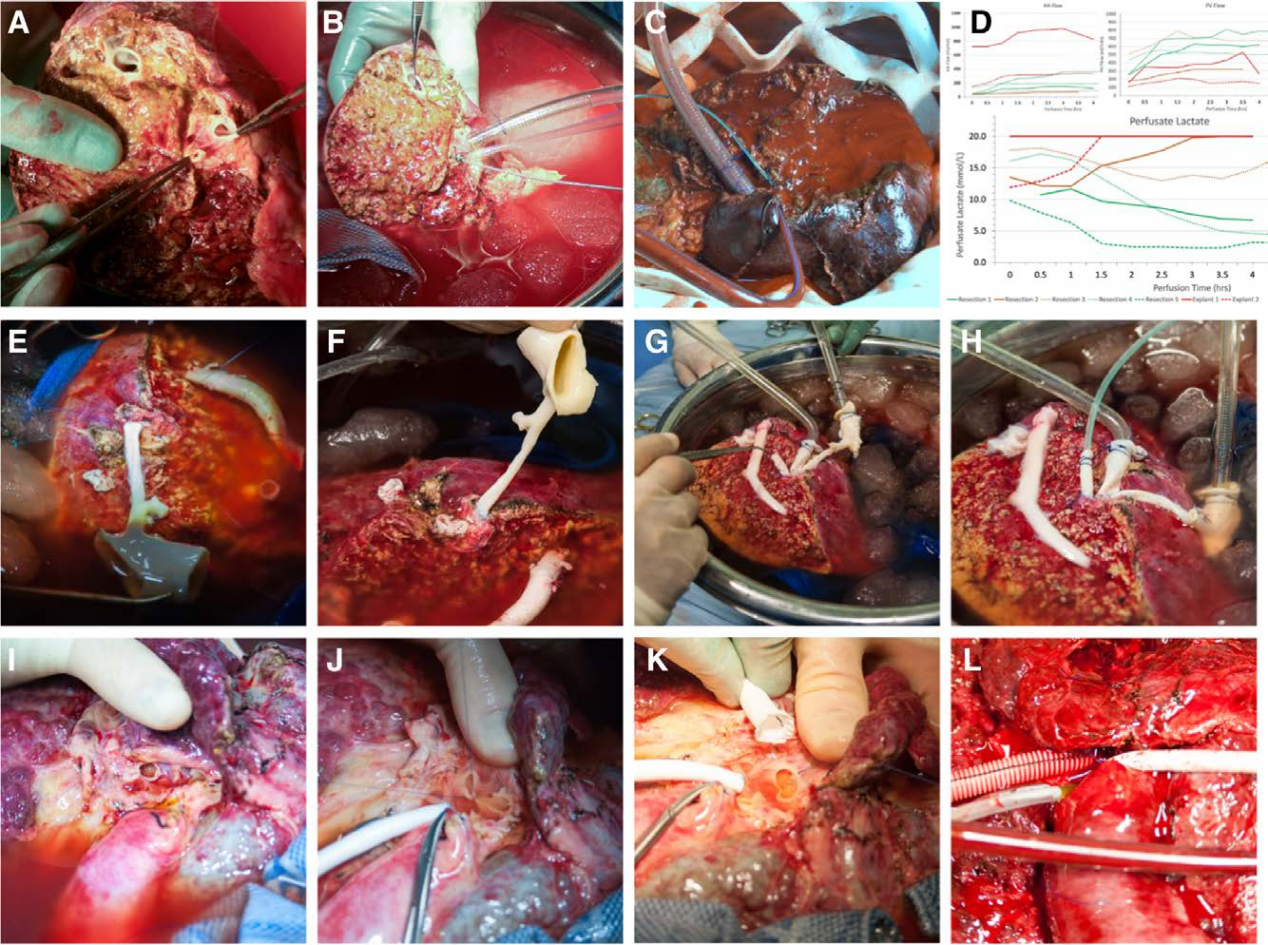
Technical aspects of liver inflow reconstruction. The figure illustrates technical details of liver cannulation and vascular inflow reconstruction. The right hepatectomy specimen (A) demonstrates short inflow vessels, with small diameter hepatic artery (pointed to by left forceps), also direct cannulation (B) might be achieved, hepatic veins outflow reconstruction was not required as the blood is collected in the device reservoir (C). The perfusion technical success might be dampened by arterial wall dissection, characterized by flows less than 100 mL per minute and rising trend in the lactate levels (D). The interposition conduit seemed beneficial to facilitate arterial (E and F), portal (G), and even bile duct (H) cannulation that can be created using donor vessels. For deceased explanted livers, the vascular inflow reconstruction is less challenging due to the larger diameter of the vessels, in particular hepatic artery (I), the portal vein reconstruction requires creation of a joint orifice (J and K). The reconstruction can be performed with synthetic vascular grafts of donor vessels, the perfusion flows in these livers require higher portal vein pressures, and the volumes through the artery were greater than in the portal vein, with livers recovering including bile production (L).

Restoring vascular inflow is the elementary requirement for liver perfusion.^2^ The hepatic artery wall dissection was critical challenge to overcome and represented the principal cause for technical failure. Direct cannulation of the artery yielded poor flows during normothermic perfusion that were overcome by inclusion of arterial conduit. Satisfactory results were achieved with a donor artery or synthetic (PTFE or Dacron) vascular graft. Achieving a suitable diameter match was difficult for both these options with donor artery being preferred for partial livers (Fig. 1). Portal inflow was easier to establish and achievable by direct cannulation.

To prevent differential portoarterial steal and promote autoregulation as well as reducing inaccuracies in the device flow readings, any significant leak from resection plane was closed on back-table or shortly after commencing perfusion.

Bile was collected via small diameter drain (pediatric feeding tube) inserted into the bile duct either on the back-table, if the bile duct was clearly identified, or after commencing the perfusion if the duct orifice could be identified by bile production.

Experiments used the liver assist device (detail) that incorporates semiopen circuit. Importantly, this device did not require hepatic venous reconstruction as the perfusate is collected and recirculated via the device reservoir.^3^ Similarly, it also allows for any bleeding from the resection plane to be recirculated. The technical success of our normothermic perfusion strategy was estimated based on the hepatic artery flows and liver ability to clearance perfusate lactate.^4^

The provided technical information is complimentary to Dr Mueller and colleagues’ report and may allow wider adoption of long-term machine perfusion models as a flexible platform with extensive research applications including pharmacology, small molecules cellular therapy, and cancer studies.
